# Harnessing neuroplasticity with psychoplastogens: the essential role of psychotherapy in psychedelic treatment optimization

**DOI:** 10.3389/fpsyt.2025.1565852

**Published:** 2025-04-08

**Authors:** Jennifer L. Jones

**Affiliations:** Department of Psychiatry and Behavioral Sciences, Medical University of South Carolina, Charleston, SC, United States

**Keywords:** psychedelics, neuroplasticity, psychoplastogens, psychedelic-assisted therapy, mental health disorders, therapeutic integration, maladaptive plasticity, cognitive flexibility

## 1 Introduction

Psychiatric disorders are marked by habitual patterns of thought, emotion, and behavior, and many mental health disorders are resistant to standard treatments (1–4). For example, symptoms of major depressive disorder include ruminative negative thoughts, pervasive sadness, and lack of goal-directed behavior (5–7). Similarly, many anxiety and trauma-related disorders are characterized by recurrent, intrusive, fear-based thoughts and patterns of avoidant behaviors (8, 9). In compulsive disorders, such as substance use disorders, obsessive-compulsive disorder, and eating disorders, individuals repeatedly engage in maladaptive behaviors and thought patterns despite negative consequences (9). Even conditions as diverse as schizophrenia may show evidence of habitual thoughts in the form of recurrent hallucinations or delusions (10). These pervasive patterns across psychiatric disorders emphasize the role of ingrained neural habits, which perpetuate symptoms and hinder lasting recovery (2, 11).

In recent years, psychedelic therapies have emerged as promising potential treatments for a range of treatment-resistant psychiatric disorders (12–19). Despite efforts to classify psychedelics by subjective effects, chemical structure, or receptor targets, previous work suggests that these categories appear to have limited relevance to therapeutic applications, as diversely grouped psychedelics have shown promise in treating depression, post-traumatic stress disorder (PTSD), and addictions, and are consequently often defined simply by their ability to induce altered states of consciousness (20). Unlike traditional pharmacotherapies, which often require weeks or months of repeated sessions to produce measurable effects, psychedelics can induce rapid and long-lasting changes following only one or a few treatments (12, 14, 15, 21). Despite their diverse molecular structures and receptor targets, psychedelics share key similarities: they generate rapid-onset therapeutic effects and produce clinical benefits that endure well beyond their metabolic clearance (22). These enduring effects suggest that neuroplasticity may serve as the unifying core mechanism underlying their therapeutic action (22). While much attention has focused on their profound and often ineffable psychoactive phenomenology, psychedelics’ neurobiological mechanisms are equally germane.

## 2 Psychoplastogens: opening a window of therapeutic opportunity

Recent research has characterized psychedelics as belonging to a newly defined class of small-molecule pharmacotherapies called psychoplastogens (23). Derived from Greek roots for mind, plasticity and generation, psychoplastogens promote neuroplasticity within 24 to 72 hours of a single administration, a stark contrast to traditional pharmacotherapies, which may take weeks to yield similar effects (23). Neuroplasticity, commonly defined as the brain’s ability to reorganize and create new neural connections, plays a vital role in adaptation, learning, and recovery (24, 25). This capacity for change manifests at multiple levels of brain organization, including alterations in connectivity between brain regions, cellular modifications, and molecular adaptations that ultimately drive behavioral level changes (26).

Human neuroimaging studies offer compelling evidence of psychedelics’ impact on neuroplasticity, with multiple reviews detailing the significant changes in brain network connectivity after their administration (27–31). For example, increases in global brain connectivity and decreased network modularity have been associated with treatment response to psilocybin and ketamine, particularly in the prefrontal cortex (PFC); these findings may correlate with enhanced cognitive flexibility (27, 29, 30, 32, 33). Further, response to both ketamine and psilocybin is associated with decreased connectivity within the default mode network (DMN) limbic nodes, correlating with reduction in habitual, self-referential thought patterns (28, 30).

At the cellular level, psychoplastogens induce changes such as increased dendrite length, spine density, synaptic number, and intrinsic excitability, typically within 24 to 72 hours of a single dose (23, 34–37). However, different psychoplastogens may promote neuroplasticity through distinct effects. For instance, in preclinical models, ketamine increases the number of dendritic spines and enhances synaptogenesis, leading to increased synaptic density and strengthened connections between neurons, particularly in the prefrontal cortex (34). Similarly, positron emission tomography (PET) studies of ketamine have demonstrated increases in synaptic density in the human brain (38). Psilocybin, on the other hand, has been shown to robustly promote dendritic complexity, increasing the length and branching of dendritic spines, which enhances connectivity and communication with neighboring neurons (23).

At the molecular level, psychoplastogens engage key signaling pathways that drive synaptic growth and neural reorganization (23). For example, ketamine acts through the NMDA receptor, leading to a surge in glutamate release, AMPA receptor activation, and subsequent stimulation of pathways such as brain-derived neurotrophic factor (BDNF) and mTOR, which support synaptogenesis and dendritic growth (22, 39–41). Similarly, MDMA modulates monoamine pathways, enhancing BDNF expression and synaptic plasticity (42, 43). Classical psychedelics like psilocybin primarily act as agonists at the 5-HT2A serotonin receptor, triggering cascades that promote synaptic signaling, increase dendritic complexity, and elevate neuroplasticity-related proteins (44). However, the implications of differing neuroplasticity effects on therapeutic efficacy are unknown.

On a behavioral level, studies have demonstrated that psychoplastogen administration can lead to significant changes in social learning, fear extinction, and adaptive behavior. For example, a Nature study found that diverse psychoplastogens enhance social learning by promoting neural plasticity in relevant brain circuits, while research on MDMA therapy has demonstrated its capacity to facilitate fear extinction learning (20, 45). Additionally, psychoplastogens have shown to reduce avoidance behaviors and promote behavioral activation (46). These behavioral changes align with observed neurobiological shifts, highlighting how psychoplastogens may translate molecular and cellular changes into meaningful clinical outcomes.

## 3 Neuroplasticity: a double-edged sword?

At face value, the ability of these compounds to catalyze neuroplasticity seems wholly beneficial. However, while neuroplasticity can allow the brain to appropriately adapt in the context of life experiences, it can also lead to maladaptive outcomes. Children with neglectful or abusive parents often develop maladaptive social tendencies, leading to difficulties in forming healthy relationships, increased vulnerability to anxiety or depression, and patterns of avoidant or aggressive behavior​ (47). This phenomenon, known as maladaptive plasticity, is also present in the reinforcement of harmful neural patterns, including those driving depression, addiction, anxiety, and PTSD (48–52).

This has important implications for psychedelic treatment design. For example, consider a hypothetical case of a combat veteran with PTSD who undergoes an unstructured psychedelic session. During the experience, the patient vividly re-experiences a traumatic memory but lacks the therapeutic support to process it effectively. Without proper integration, the session may inadvertently reinforce the brain’s fear circuits, leading to heightened hypervigilance and emotional reactivity. Instead of alleviating symptoms, the session could exacerbate the maladaptive patterns and further diminish adaptive regulation of fear responses.

This risk of maladaptive plasticity is not unique to PTSD. In substance use disorders, drug misuse strengthens reward pathways (51). As these circuits adapt, individuals experience heightened cravings and diminished sensitivity to natural rewards, perpetuating the addictive cycle (51). Over time, this plasticity can lead to compulsive behavior patterns that are resistant to change, even in the face of severe consequences (53, 54). Maladaptive plasticity also plays a role in chronic pain, where the brain continues to perceive pain signals even after the initial injury has healed, leading to a cycle of fear and avoidance behaviors (55–57). Consequently, neuroplasticity can be conceptualized as a double-edged sword. While it enables growth, learning, and recovery, it also underscores the importance of structured interventions to ensure that changes are beneficial.

## 4 Guiding neuroplasticity: lessons from neurorehabilitation

Given that neuroplasticity can lead to either adaptive or maladaptive changes, how can clinicians selectively guide these effects toward positive outcomes? Neurorehabilitation models provide a constructive example of how neuroplasticity can be harnessed to selectively improve functioning and effect desired clinical outcomes. For example, after a stroke, spontaneous neuroplastic changes may occur, but are often insufficient for full recovery of lost functions (58, 59). Through targeted rehabilitation, therapy aims to retrain and reorganize the brain’s neural pathways to regain these abilities (26, 60). Physical therapy, for instance, focuses on repetitive movement to stimulate neuroplastic changes in the motor cortex, gradually rebuilding strength (60, 61). Speech therapy helps patients regain communication skills by stimulating the brain’s language centers (62). Occupational therapy focuses on adaptive functional recover of daily fine motor tasks, while cognitive therapy targets memory and attention deficits (63, 64). Without such synergistic and targeted therapeutic interventions, neuroplasticity may lead to maladaptive outcomes, such as learned nonuse of the affected limb or inefficient compensatory strategies (65). Structured, goal-directed therapy provides the necessary guidance to optimally channel neuroplastic changes.

## 5 Characterizing the contributions of therapy in shaping positive psychiatric outcomes

Both historical and contemporary clinical literature underscore the role of psychotherapy in maximizing the therapeutic potential of neuroplasticity (66–71). Pioneering work on psychedelic therapies emphasized the necessity of structured therapeutic frameworks to translate the experiential insights of psychedelics into lasting behavioral change (69, 70). Such work has highlighted how psychotherapy not only supported safety and integration but also actively guided individuals toward adaptive change (72–74).

Psychoplastogen-assisted psychotherapeutic methods have differed across studies. For ethical conduct of research, a minimum standard includes basic preparatory psychoeducation about the nature of the psychoactive experience (75). However, a majority of psychedelic trials include additional preparatory therapeutic support, offering guidance on navigating non-ordinary states of consciousness, especially during intense or challenging emotions (13). Preparatory sessions are subsequently followed by one or more medication sessions, which includes at a minimum, supervision for safety if not psychological support or psychotherapy (13).

Post-medication therapy sessions, when included, often differ across clinical trials (13, 67, 76, 77). For instance, a few ketamine-assisted psychotherapy trials have utilized specific manualized psychotherapy platforms (e.g. motivational enhancement therapy, mindfulness-based therapy, and cognitive behavioral therapy) (78–82). Additionally, many of the treatment-based psychoplastogen clinical trials include post-medication integratory psychotherapy sessions (13). These sessions are generally multimodal, drawing from a wide variety of therapeutic lineages, akin to the multimodal style commonly reported by practicing clinical therapists (13, 83), with the goal of translating psychological insights into positive actionable changes in daily life (84). For instance, the psychodynamic approach to therapy might be deliberately utilized to uncover unconscious patterns, emotions, and conflicts rooted in past experiences, this technique may be flexibly employed to help patients explore and process unconscious material and insights that emerges during the psychedelic experience (85–87). Cognitive-behavioral therapy (CBT) techniques may be used to help individuals identify and restructure negative thought patterns discovered during sessions (88–90). Acceptance and commitment therapy (ACT) techniques can encourage patients to develop greater psychological flexibility, accept difficult emotions, and commit to meaningful action aligned with their values (90–92). Elements of motivational enhancement therapy (MET) may be integrated to help patients strengthen their intrinsic motivation for change and align their insights with concrete behavioral goals (93). Behavioral activation therapy techniques, may be utilized to encourage patients to engage in positive, rewarding activities that align with their values, while mindfulness practices may be employed to enhance self-awareness and emotional acceptance (94, 95).

## 6 Discussion

Building on established psychotherapy techniques, psychedelic-assisted therapies offer a novel and promising approach to mental health treatment by harnessing the power of neuroplasticity. By opening up a “window of opportunity” for change, psychoplastogen-assisted therapy can help individuals deliberately break free from entrenched patterns of thought, emotion, and behavior, allowing a path forward towards enduring healing and growth. This process is reminiscent of critical periods in early development, where the brain is particularly sensitive to learning and environmental influences.

**Figure 1:**
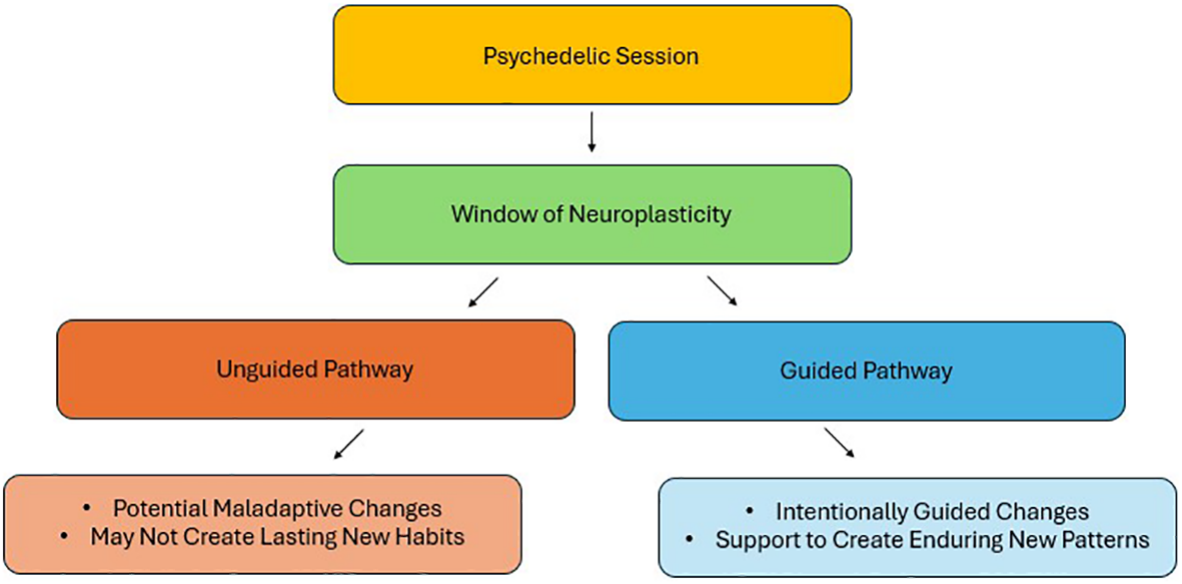
Divergent pathways from the neuroplasticity window opened by psychedelics: adaptive vs. maladaptive outcomes.

However, neuroplasticity is not inherently beneficial and requires careful therapeutic guidance to avoid maladaptive outcomes. Just as targeted, multimodal therapies are necessary to guide neuroplastic changes toward recovery following a stroke, psychotherapy is essential to direct psychedelic-induced neuroplasticity toward positive outcomes. Integrated psychotherapeutic approaches create a framework to translate the insights gained during psychedelic experiences into tangible behavioral and cognitive changes. Without therapeutic support however, the neuroplastic changes induced by psychedelics may be maladaptive, or not be properly consolidated, potentially limiting the durability and efficacy of treatment (**Figure 1**).

As psychoplastogen treatments increasingly expand into clinical practice, personalized therapy approaches will likely be tailored to an individual’s unique clinical profile, such as incorporating motivational enhancement techniques for individuals with substance use disorders, leveraging cognitive-behavioral strategies or behavioral activation techniques to target major depressive disorder, or intentionally using somatic techniques to target dissociative tendencies. Consequently, therapist training and competency assessments may prove a better regulatory focus than rigid therapeutic protocols. Ultimately however, while psychedelics may open a window to neuroplastic change, it is the guiding hand of psychotherapy that will ensure the path chosen leads to positive mental health transformation.
